# Mitochondrial Dysfunction as a Targetable Vulnerability in Gastric Cancer: From Carcinogenesis to Apoptosis (A Narrative Review)

**DOI:** 10.7150/jca.131081

**Published:** 2026-08-10

**Authors:** Hsi-Lung Hsieh, Ming-Chin Yu, Hui-Ching Tseng, Yi-Hsuan Wu, Tzu-Hao Huang, Ming-Ming Tsai

**Affiliations:** 1 Graduate Institute of Health Industry Technology, Center for Drug Research and Development, College of Human Ecology, Chang Gung University of Science and Technology, Taoyuan 333, Taiwan; 2 Department of Neurology, Chang Gung Memorial Hospital, Taoyuan 333, Taiwan; 3 Department of Chemical Engineering, R&D Center of Biochemical Engineering Technology, Ming Chi University of Technology, New Taipei City 301, Taiwan; 4 Department of General Surgery, New Taipei Municipal TuCheng Hospital, New Taipei 236, Taiwan; 5 College of Medicine, Chang Gung University, Taoyuan 333, Taiwan; 6 Department of General Surgery, Chang Gung Memorial Hospital, Taoyuan 333, Taiwan; 7 Department of Nursing, Division of Basic Medical Sciences, Chang Gung University of Science and Technology, Taoyuan 333, Taiwan

**Keywords:** gastric cancer, mitochondrial dysfunction, mitochondrial biomarkers, metabolic reprogramming, apoptosis, natural compounds

## Abstract

Gastric cancer (GC) remains a major global health burden and is associated with high mortality worldwide. Current treatment integrates surgery, perioperative or systemic chemotherapy, molecularly targeted therapy, and immune checkpoint blockade; however, efficacy is frequently limited by intratumoral heterogeneity, metabolic plasticity, and therapeutic resistance. Mitochondria, as central regulators of cellular bioenergetics, redox homeostasis, and apoptotic signaling, are profoundly altered in GC. Tumor cells frequently exhibit enhanced aerobic glycolysis accompanied by suppressed oxidative phosphorylation, reflecting mitochondrial metabolic remodeling. Accumulating evidence indicates that mitochondrial dysfunction actively contributes to tumor initiation, progression, and therapeutic resistance in GC. Importantly, these mitochondrial alterations also create metabolic vulnerabilities that may be therapeutically exploitable. Both conventional chemotherapeutic agents and herbal-derived natural compounds have been reported to induce mitochondrial stress responses, including excessive reactive oxygen species (ROS) accumulation, mitochondrial membrane potential loss, and activation of mitochondria-dependent intrinsic apoptotic pathways. In this narrative review, we summarize the major molecular mechanisms underlying mitochondrial dysfunction in GC, highlight mitochondrial-associated prognostic biomarkers, and discuss emerging therapeutic strategies targeting mitochondrial pathways. Collectively, these insights emphasize mitochondrial dysfunction as a candidate therapeutic vulnerability and support further investigation of mitochondria-centered therapeutic strategies for GC.

## Introduction

Gastric cancer (GC) remains one of the most prevalent malignancies worldwide and continues to impose a substantial global health burden. The disease shows particularly high incidence and mortality rates in East Asia, Eastern Europe, and parts of South America 1, 2. Despite advances in surgical techniques, perioperative management, and systemic therapies, the prognosis of advanced GC remains poor, with five-year overall survival (OS) rates generally below 30% 3. Current standard-of-care treatment is stage- and biomarker-dependent and includes surgery with perioperative therapy for resectable disease, systemic chemotherapy, immune checkpoint inhibitors, and targeted therapies directed against HER2 and VEGF/VEGFR pathways 4, 5. However, the clinical efficacy of these therapeutic strategies is frequently limited by the rapid emergence of drug resistance, intratumoral heterogeneity, metabolic plasticity, and dysregulation of apoptotic signaling pathways, resulting in transient or suboptimal therapeutic responses 6-8.

Mitochondria play central roles in cellular bioenergetics, redox homeostasis, calcium signaling, and apoptosis regulation, and function as master regulators of cellular stress signaling and cell fate determination 9. In this review, mitochondria-targeted agents refer to compounds specifically designed to accumulate within mitochondria or directly disrupt mitochondrial structures or functions, whereas mitochondria-associated agents refer to conventional drugs whose primary pharmacological targets are not mitochondria but that exert secondary effects on mitochondrial function. This distinction helps clarify the mechanistic basis of mitochondria-related therapeutic strategies discussed throughout this review.

Accumulating evidence indicates that mitochondrial dysfunction is a prominent and recurrent feature of GC biology. GC cells commonly exhibit suppressed oxidative phosphorylation (OXPHOS) and enhanced aerobic glycolysis driven by the Warburg effect, reflecting profound mitochondrial metabolic remodeling that supports tumor growth and metabolic plasticity 10. GC cells commonly exhibit metabolic reprogramming, increased mitochondrial stress, and dysregulation of apoptosis-related signaling 7, 11-13. These mitochondrial alterations not only reflect extensive metabolic reprogramming but also actively promote tumor progression by engaging mitochondrial retrograde signaling pathways that modulate nuclear gene expression 8, 12, 14, 15. Through mitochondria-nucleus communication, HIF-1α signaling can be activated, whereas p53- and PTEN-mediated metabolic regulation may be impaired, thereby contributing to malignant progression and therapeutic resistance in GC 12-17.

Mitochondrial genome instability represents another important layer of mitochondrial dysregulation in GC. Mitochondrial DNA (mtDNA) alterations—including point mutations, deletions, insertions, and copy-number variations—are frequently detected in GC tissues and have been associated with impaired mitochondrial respiration, reduced ATP generation, and excessive oxidative stress 18, 19. Elevated mitochondrial ROS further amplify mitochondrial damage and initiate a self-propagating cycle of oxidative stress and metabolic dysfunction that contributes to tumor progression and metastasis 8, 20.

Altered mtDNA copy number and mtDNA instability have been linked to cancer progression and may have potential utility as mitochondria-related biomarkers 21.

Oncogenic activation and tumor-suppressor inactivation further reinforce mitochondrial metabolic reprogramming and metabolic plasticity in cancer cells 6, 11, 12. In parallel, hypoxia-inducible factor-1α (HIF-1α) acts as a central metabolic regulator that coordinates mitochondrial metabolism and redox balance under hypoxic conditions. Through this mechanism, tumor cells can adapt to hypoxic stress, evade ROS-induced apoptosis, and promote angiogenesis, invasion, and metabolic adaptation 11, 15, 22.

**Figure 1:**
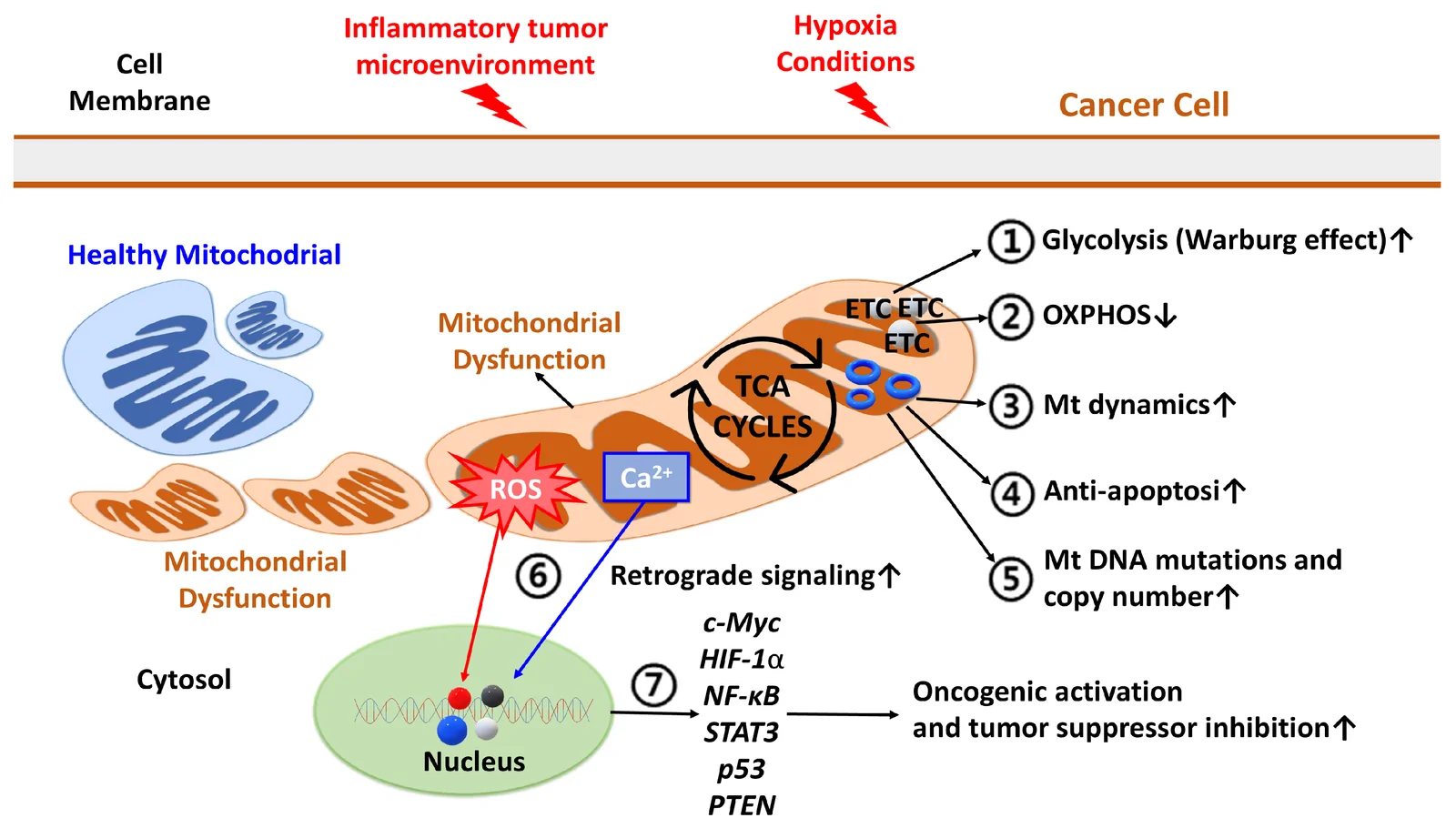
** Mitochondrial dysfunction as a central pathogenic mechanism and targetable vulnerability in GC.** GC cells exhibit extensive mitochondrial alterations, including suppressed oxidative phosphorylation (OXPHOS), enhanced aerobic glycolysis driven by the Warburg effect, mitochondrial genome instability, redox imbalance, dysregulated mitochondrial dynamics, and impaired mitochondria-dependent apoptosis. Disruptions in mitochondrial calcium (Ca²⁺) handling, excessive accumulation of mitochondrial reactive oxygen species (mtROS), imbalance in AMP/ATP ratios, and altered NADH/NAD+ redox balance further trigger mitochondrial retrograde signaling to the nucleus, leading to sustained transcriptional reprogramming of nuclear genes. These mitochondria-derived signals subsequently activate key oncogenic pathways, including hypoxia-inducible factor-1α (HIF-1α), MYC, nuclear factor-κB (NF-κB), and signal transducer and activator of transcription 3 (STAT3), while suppressing tumor-suppressor pathways such as p53 and PTEN. Collectively, these interconnected processes reinforce glycolytic dependency and promote tumor survival, invasion, angiogenesis, and therapeutic resistance. Thus, mitochondrial dysfunction acts both as a central pathogenic mechanism and as a candidate therapeutically exploitable vulnerability in GC. **Abbreviations:** OXPHOS: oxidative phosphorylation; mtROS: mitochondrial reactive oxygen species; ΔΨm: mitochondrial membrane potential; NADH: reduced nicotinamide adenine dinucleotide; NAD+: oxidized nicotinamide adenine dinucleotide; NF-κB: nuclear factor kappa B.

Imbalances in mitochondrial calcium (Ca²⁺) handling, reactive oxygen species (ROS) levels, AMP/ATP ratios, and reduced nicotinamide adenine dinucleotide (NADH) redox status can trigger mitochondrial retrograde signaling, leading to sustained nuclear transcriptional reprogramming that facilitates tumor survival and metabolic adaptation in GC 6, 12, 16. Under hypoxic conditions, mitochondria-derived ROS further activate the HIF-1α pathway, thereby reinforcing glycolytic dependence and malignant progression 14, 15, 22. A schematic overview of the interconnected roles of mitochondrial metabolism, redox imbalance, mitochondrial dynamics, and retrograde signaling in gastric carcinogenesis is shown in Figure 1.

Concurrently, growing evidence indicates that chemical agents, mitochondria-directed systems, and natural compounds can exert anti-GC effects through mitochondria-associated or mitochondria-targeted mechanisms. Representative examples include 17-dimethylaminoethylamino-17-demethoxygeldanamycin (17-DMAG), topotecan, doxorubicin (DOX) derivatives, and mitochondria-targeting peptides such as Mito-FF, as well as herbal-derived compounds including baicalein, curcumin, shikonin, and resveratrol (Tables 3 and 4). Depending on the agent, reported mitochondrial effects include mitochondrial membrane potential (ΔΨm) collapse, mitochondrial ROS accumulation, cytochrome c release, dissociation of hexokinase 2 (HK2) from the voltage-dependent anion channel (VDAC), or mitochondrial fission, ultimately promoting mitochondria-dependent intrinsic apoptosis in GC cells 9, 23, 24. Importantly, targeting mitochondrial vulnerabilities may provide opportunities for rational combination strategies with conventional chemotherapy by exploiting cancer-specific metabolic dependencies while warranting further preclinical and clinical validation.

This review provides a comprehensive overview of mitochondrial dysfunction in GC, focusing on mitochondrial metabolism, redox regulation, mitochondrial dynamics, and apoptosis signaling pathways. We further summarize mitochondrial-associated biomarkers and therapeutic strategies targeting mitochondrial pathways, including conventional chemotherapeutic agents and natural compounds, and discuss emerging translational perspectives for the further investigation of mitochondria-centered therapeutic approaches in GC.

## Search Strategy and Study Selection

To provide a comprehensive overview of mitochondrial dysfunction in GC, relevant studies were identified through searches of major biomedical databases, including PubMed, Web of Science, and Scopus. The search strategy used combinations of the following keywords: (“gastric cancer” OR “gastric carcinoma”) AND (“mitochondria” OR “mitochondrial dysfunction” OR “mitochondrial apoptosis” OR “mitochondrial metabolism” OR “mitochondrial biomarkers”).

Studies published in English from database inception through June 2026 were considered. The literature search was last updated in June 2026. Priority was given to original research articles and review articles investigating mitochondrial metabolism, mitochondria-associated and mitochondria-targeted therapeutic strategies, and mitochondrial biomarkers in GC. Both experimental studies (*in vitro* and *in vivo*) and clinical investigations were included to provide an integrated overview of current mechanistic insights and translational progress. ChatGPT (OpenAI) was used solely for English-language editing of selected passages and was not used for literature retrieval, study selection, data extraction, evidence synthesis, or scientific interpretation.

## Part I. Metabolic Reprogramming (Warburg Effect) and Mitochondrial Dysfunction-Related Biomarkers in Gastric Carcinogenesis

Over the past decade, accumulating evidence has identified metabolic reprogramming as a central driver of mitochondrial dysfunction in GC. Even under normoxic conditions, GC cells preferentially rely on aerobic glycolysis rather than oxidative phosphorylation (OXPHOS), a metabolic phenotype widely known as the Warburg effect 7. This metabolic shift suppresses mitochondrial respiration and electron transport chain (ETC) activity, reduces ATP production efficiency, and is frequently accompanied by mitochondrial genomic alterations, including mitochondrial DNA (mtDNA) mutations and depletion 19.

As a consequence, mitochondrial dysfunction promotes excessive accumulation of mitochondrial reactive oxygen species (mtROS), destabilization of the mitochondrial membrane potential (ΔΨm), and dysregulation of mitochondrial fusion-fission dynamics, particularly dynamin-related protein 1 (DRP1)-mediated mitochondrial fission. These alterations activate mitochondrial retrograde signaling pathways that reprogram nuclear gene expression and facilitate oncogenic transformation 14, 25-28. Through mitochondria-nucleus communication, oncogenic signaling pathways such as hypoxia-inducible factor-1α (HIF-1α) and signal transducer and activator of transcription 3 (STAT3) become persistently activated, whereas tumor-suppressor-mediated metabolic regulatory pathways involving p53 and PTEN are functionally impaired 16, 29, 30. Collectively, these mitochondrial abnormalities enhance glycolytic dependency, promote tumor aggressiveness, and contribute to therapeutic resistance in GC.

Dysregulated expression or activity of several mitochondria-associated metabolic, antioxidant, mitochondrial-dynamics, and apoptosis-regulating proteins has been investigated in relation to GC biology, treatment response, or clinical outcomes. The strength and direction of the evidence vary by biomarker. Representative biomarkers include hexokinase 2 (HK2), voltage-dependent anion channel 1 (VDAC1), pyruvate dehydrogenase kinases (PDK1/4), pyruvate dehydrogenase (PDH), the mitochondrial complex IV-associated protein NDUFA4, fumarate hydratase (FH), the mitochondrial biogenesis regulator peroxisome proliferator-activated receptor gamma coactivator 1 alpha (PGC-1α), redox regulators including sirtuin 3 (SIRT3), superoxide dismutase 2 (SOD2), and mitochondrial glutathione (mtGSH), regulators of mitochondrial dynamics and quality control such as dynamin-related protein 1 (DRP1), PTEN-induced kinase 1 (PINK1), the E3 ubiquitin ligase Parkin (PARK2), and mitochondrial transcription factor A (TFAM), and the apoptosis regulator B-cell lymphoma 2 (BCL-2). Their canonical functions, detection methods, reported clinical significance, and evidence levels are summarized in Table 1.

Importantly, several mitochondrial biomarkers summarized in Table 1 have begun to demonstrate potential translational relevance in clinical GC research. HK2 and SIRT3 have been evaluated in clinical cohorts and linked to tumor aggressiveness or survival 31-34. BCL-2 is mechanistically relevant to chemosensitivity, but its association with overall survival is inconsistent and context-dependent 35-37.

In clinical research settings, these biomarkers are most commonly evaluated by immunohistochemistry (IHC) in tumor tissues 31-36, whereas emerging approaches also include serum-based biomarkers such as fumarate hydratase autoantibodies (FH-Ab) 38, circulating mitochondrial DNA assays, and functional mitochondrial analyses.

From a translational perspective, mitochondrial biomarkers may provide valuable tools for patient stratification and risk prediction, particularly in identifying GC patients who exhibit strong mitochondrial metabolic dependence or redox vulnerability. Such biomarkers may therefore facilitate patient stratification and the further exploration of mitochondria-centered therapeutic strategies, including the rational application of mitochondria-associated chemotherapeutic agents and emerging mitochondria-targeted therapies in GC.

Although mitochondrial biomarkers such as BCL-2, HK2, and SIRT3 have shown prognostic or mechanistic relevance in GC, their routine clinical implementation remains challenging. Previous studies have reported associations between HK2 overexpression and unfavorable outcomes in GC or digestive system tumors 31, 34, 39. In addition, altered SIRT3 expression and BCL-2 expression have been associated with GC prognosis or chemoresistance, although the direction and strength of these associations are not uniform across studies 35-37. These findings remain influenced by differences in antibody selection, immunohistochemical scoring systems, patient cohorts, and cutoff definitions. At present, unlike established or emerging clinically actionable biomarkers such as HER2, MMR/MSI, CLDN18.2, and PD-L1, BCL-2, HK2, and SIRT3 have not been incorporated into standardized routine GC patient-stratification algorithms 40. Similarly, circulating mtDNA-related approaches and functional mitochondrial assays, including oxygen consumption rate, mitochondrial membrane potential, ATP production, and mitochondrial ROS measurement, remain largely research-oriented tools rather than validated clinical assays, partly because of technical variability, sample-dependent limitations, cost, and the lack of harmonized analytical platforms 41. Therefore, the clinical application of mitochondrial biomarkers in precision oncology will require standardized cutoff values, assay harmonization, prospective validation, and integration with established clinicopathological and molecular classification systems.

### 1. Reprogrammed glycolysis (Warburg effect): HK2-VDAC signaling axis

Hexokinase 2 (HK2) is frequently overexpressed in GC and functions as a key rate-limiting enzyme in glycolysis, catalyzing the conversion of glucose to glucose-6-phosphate. Upregulation of HK2 drives metabolic reprogramming toward the Warburg phenotype and supports tumor growth by sustaining high glycolytic flux 31, 34. At the mitochondrial level, HK2 interacts with voltage-dependent anion channel 1 (VDAC1) on the outer mitochondrial membrane (OMM), forming the HK2-VDAC signaling axis that coordinates glycolytic metabolism with mitochondrial function 42, 43.

This interaction not only enhances ATP production through glycolysis but also stabilizes mitochondrial membrane integrity and suppresses mitochondria-dependent apoptotic signaling. By binding to VDAC1, HK2 prevents cytochrome c release and inhibits mitochondrial outer membrane permeabilization (MOMP), thereby conferring resistance to apoptosis in GC cells. In addition, HK2 exhibits dynamic subcellular localization between the cytosol and mitochondria, reflecting its role as a metabolic regulator responding to cellular energetic demands.

Experimental disruption of mitochondrial HK2 localization has been shown to impair glycolytic metabolism, increase mitochondrial stress, and sensitize GC cells to mitochondrial stress-induced apoptosis 44. Clinically, elevated HK2 expression in GC tissues is significantly associated with aggressive tumor behavior and reduced overall survival (OS), highlighting the HK2-VDAC axis as a clinically relevant prognostic biomarker and a candidate mitochondria-centered therapeutic target 31, 34.

### 2. Remodeling of TCA cycle and ETC/OXPHOS enzymes

#### PDK-PDH metabolic gate

Mitochondrial pyruvate metabolism represents a critical metabolic checkpoint regulating the balance between glycolysis and oxidative phosphorylation. In GC, dysregulation of the pyruvate dehydrogenase kinase (PDK)-pyruvate dehydrogenase (PDH) axis has been consistently reported. Upregulation of PDK1 inhibits PDH activity through phosphorylation, thereby preventing pyruvate entry into the tricarboxylic acid (TCA) cycle and shifting cellular metabolism toward glycolysis 45.

Elevated PDK1 expression has been detected in GC tissues and has been associated with tumor progression and poor prognosis 45. Mechanistically, PDK1 phosphorylates and inhibits PDH, thereby restricting pyruvate entry into the tricarboxylic acid cycle and favoring glycolytic metabolism. Separately, miR-21-5p-mediated suppression of PDHA1 has been shown to enhance glycolysis and promote GC progression 46. PDK4 is overall downregulated in GC datasets and has been implicated in mitochondrial metabolic remodeling and immune regulation. However, the reported associations of PDK4 expression with tumor stage and patient survival appear context-dependent and require further validation 47. Together, these findings identify the PDK-PDH metabolic gate as an important regulatory node linking mitochondrial metabolism to gastric carcinogenesis.

#### ETC and TCA cycle enzyme remodeling

Extensive remodeling of the ETC and oxidative phosphorylation machinery represents another hallmark of mitochondrial dysfunction in GC. NDUFA4, a mitochondrial complex IV-associated protein, contributes to ETC function and mitochondrial respiration. NDUFA4 is highly expressed in GC tissues, and its elevated expression has been associated with poor prognosis. Functional studies indicate that NDUFA4 promotes glycolytic and oxidative metabolism, cell proliferation, and tumor growth, whereas NDUFA4 knockdown suppresses these phenotypes 48, 49.

Fumarate hydratase (FH) catalyzes the conversion of fumarate to malate and is essential for TCA-cycle function. In GC experimental models, suppression of FH activity increased the efficacy of cisplatin-mediated chemotherapy, supporting a treatment-response role for tissue/cellular FH rather than an established prognostic association 50. Separately, serum FH autoantibodies (FH-Ab) have shown stage-specific alterations in patients with GC; reduced serum FH-Ab levels were associated with poorer outcomes and shorter survival 38. Tissue FH and serum FH-Ab should therefore be interpreted as distinct biomarkers with different sample types, detection methods, and evidence levels.

At the transcriptional level, peroxisome proliferator-activated receptor gamma coactivator-1 alpha (PGC-1α) functions as a master regulator of mitochondrial biogenesis and oxidative metabolism. Dysregulation of PGC-1α has been implicated in metabolic remodeling across digestive system malignancies; however, its direction of change and independent prognostic significance in GC remain insufficiently established 51. Accordingly, PGC-1α should currently be regarded as a mechanistically relevant candidate rather than a clinically validated GC biomarker.

### 3. Altered mitochondrial redox regulation

Disruption of mitochondrial redox homeostasis represents a key component of mitochondrial dysfunction in GC. Sirtuin-3 (SIRT3), a mitochondrial NAD⁺-dependent deacetylase localized in the mitochondrial matrix, acts as a central regulator of oxidative phosphorylation, fatty acid oxidation, and antioxidant defense. Reduced SIRT3 expression in GC tissues leads to excessive mtROS accumulation, activation of HIF-1α signaling, and reinforcement of the Warburg phenotype 32, 33. Clinically, decreased SIRT3 levels are associated with aggressive tumor behavior and unfavorable prognosis in GC cohorts.

Manganese-dependent superoxide dismutase (MnSOD/SOD2) is a primary mitochondrial antioxidant enzyme responsible for detoxifying superoxide radicals within the mitochondrial matrix. In human GC tissue, altered MnSOD expression has been associated with the mode of tumor invasion 52, whereas experimental SOD2 modulation affects proliferation and invasion in GC models 53. These data support biological relevance, but an independent prognostic role remains to be validated.

In addition to enzymatic antioxidant systems, mitochondrial glutathione (mtGSH) represents the principal non-enzymatic redox buffer within the mitochondrial matrix. Depletion of mtGSH disrupts mitochondrial redox balance, promotes mtROS accumulation, and can enhance mitochondrial dysfunction across experimental disease and cancer models. However, the cited evidence is not GC-specific; therefore, mtGSH should be regarded as a mechanistic mitochondrial redox factor rather than a validated GC prognostic or therapeutic biomarker 54.

### 4. Dysregulated mitochondrial dynamics and quality control

Mitochondrial dynamics and quality control mechanisms are essential for maintaining mitochondrial integrity and cellular homeostasis. Dysregulation of these processes has been increasingly recognized as a contributor to GC progression.

Dynamin-related protein 1 (DRP1/DNM1L) is a central GTPase that regulates mitochondrial fission through recruitment from the cytosol to the OMM, where it interacts with receptors such as MFF, FIS1, MID49, and MID51 55, 56. In GC cell models, DRP1-dependent mitochondrial fission has been linked to adriamycin resistance through BATF2/p53/ERK signaling 55. These findings support a mechanistic role for DRP1 in treatment resistance, but its independent prognostic value in patients with GC remains insufficiently established.

PINK1/Parkin-dependent mitophagy appears to exert context-dependent effects in GC. Metformin-induced activation of this pathway has been associated with cisplatin resistance 57, whereas impaired mitophagy under hypoxic conditions can enhance mtROS/HIF-1α signaling and tumor aggressiveness 58. These findings indicate that the biological consequences of PINK1/Parkin signaling depend on treatment and microenvironmental context.

Another key regulator of mitochondrial homeostasis is mitochondrial transcription factor A (TFAM), which governs mtDNA transcription and replication. In AGS cells, experimental TFAM knockdown reduced mtDNA copy number and mitochondrial respiration while increasing glycolytic dependence and cell migration. In the clinical component of the cited study, poorer outcomes were associated with low mtDNA copy number and the mtDNA D310 mutation rather than with elevated TFAM expression. Therefore, the direction and prognostic significance of TFAM expression in clinical GC tissues remain insufficiently established 59.

### 5. Mitochondria-dependent apoptosis

Mitochondria-dependent apoptosis represents a crucial tumor-suppressive mechanism frequently dysregulated in cancer. B-cell lymphoma 2 (BCL-2) is a prototypical anti-apoptotic protein predominantly localized to the outer mitochondrial membrane, where it inhibits cytochrome c release and prevents activation of the intrinsic apoptotic pathway.

In GC, BCL-2 expression has been associated with altered sensitivity to chemotherapeutic agents such as cisplatin and 5-fluorouracil 36. However, its prognostic significance is inconsistent: a meta-analysis found no significant overall association with OS, although favorable associations were reported in Asian subgroup analyses 35, 37. BCL-2 is therefore mechanistically relevant to apoptosis resistance and treatment response but should not be presented as an established poor-prognosis biomarker.

#### Integrative Perspective

Collectively, coordinated dysregulation of glycolysis-mitochondrial coupling, oxidative phosphorylation, TCA cycle integrity, redox homeostasis, mitochondrial dynamics, mitochondrial genome stability, and apoptosis regulation cooperatively drives metabolic reprogramming, tumor aggressiveness, and poor clinical outcomes in GC. Together, these mitochondria-associated molecular alterations highlight mitochondrial dysfunction as a unifying pathogenic axis and a candidate therapeutic vulnerability in gastric carcinogenesis, as summarized in Table 1.

## Part II. Mitochondria-Associated and Mitochondria-Targeted Anticancer Mechanisms of Chemotherapeutic Agents in GC

Mitochondrial dysfunction plays a dual role in GC, serving not only as a driving force for tumor initiation and progression but also as a critical source of therapeutic vulnerability. Although mitochondrial metabolic reprogramming, redox imbalance, and suppression of mitochondria-dependent apoptosis promote tumor cell survival, malignant progression, and therapeutic resistance, these same abnormalities render GC cells particularly susceptible to mitochondrial stress. Therefore, understanding the dual roles of mitochondrial dysfunction in gastric carcinogenesis and treatment response is essential for elucidating GC biology and for informing the development of therapeutic strategies.

In recent years, mitochondria have emerged as a candidate therapeutic vulnerability in GC. Increasing evidence indicates that the cytotoxic effects of several conventional chemotherapeutic agents extend beyond nuclear DNA damage and cell-cycle arrest to include mitochondria-associated stress responses. In addition, many anticancer agents exert part of their antitumor activity through mitochondria-associated mechanisms. By disrupting mitochondrial bioenergetics, redox homeostasis, mitochondrial membrane potential, and mitochondrial dynamics, these agents activate intrinsic mitochondria-dependent apoptotic pathways and ultimately induce GC cell death (Table 3).

Based on current experimental evidence, several pharmacological agents and experimental interventions exert anticancer activity in GC through mitochondria-associated or mitochondria-targeted mechanisms (Table 3). Although these interventions belong to different classes, their cytotoxic effects can include mitochondrial oxidative stress, disruption of mitochondrial membrane potential (ΔΨm), mitochondrial outer membrane permeabilization (MOMP), or activation of intrinsic apoptotic signaling. The occurrence and extent of these mitochondrial perturbations vary by agent and experimental model.

To avoid mechanistic overgeneralization, agents that affect mitochondrial function should be distinguished as mitochondria-targeted agents or mitochondria-associated agents. Mitochondria-targeted agents are compounds or delivery systems designed to preferentially accumulate within mitochondria or directly interact with mitochondrial membranes, respiratory-chain components, mitochondrial metabolic enzymes, or mitochondrial apoptosis regulators. In contrast, mitochondria-associated agents primarily act through non-mitochondrial targets but secondarily induce mitochondrial stress, including ROS accumulation, mitochondrial membrane potential collapse, ATP depletion, cytochrome c release, or activation of intrinsic apoptosis. This distinction is important because direct mitochondrial targeting and indirect mitochondrial perturbation require different validation methods, pharmacological considerations, and translational strategies 60, 61.

Mechanistically, several chemotherapeutic agents exert anticancer effects by inducing mitochondrial oxidative stress and activating intrinsic apoptotic pathways in GC cells. The HSP90 inhibitor 17-DMAG disrupts intracellular oxidant-antioxidant balance, leading to excessive ROS accumulation, suppression of antioxidant defenses, and activation of apoptosis in GC cells 62. These findings identify redox imbalance as an important mediator of 17-DMAG-induced cytotoxicity.

Anthracycline-based chemotherapy also exerts prominent mitochondrial effects in GC. DOX-associated mitochondrial apoptosis can be potentiated by mitochondrial stress-modulating compounds. For example, Parameritannin A-2 enhances doxorubicin-induced mitochondria-dependent apoptosis by inhibiting the PI3K/AKT, ERK1/2, and p38 pathways in GC cells 63.

In addition, the cathepsin B-cleavable DOX prodrug Ac-Phe-Lys-PABC-DOX (PDOX) has been reported to induce mitochondria-associated oxidative stress and apoptosis in MGC-803 cells 64.

The topoisomerase I inhibitor topotecan has also been reported to induce mitochondrial cytotoxicity in GC. Experimental studies demonstrate that topotecan triggers apoptosis through oxidative stress-related mechanisms, leading to excessive reactive oxygen species (ROS) accumulation, mitochondrial membrane depolarization (ΔΨm loss), and activation of intrinsic apoptotic pathways in GC cells 65, 66.

The widely used chemotherapeutic agent 5-fluorouracil (5-FU) has also been associated with increased apoptosis in GC. Specifically, preoperative 5-FU administration was associated with an increased apoptotic fraction in resected tumor tissues from patients with advanced GC 67. However, this clinical tissue study did not directly assess mitochondrial ROS production, mitochondrial membrane depolarization, or other mitochondrial functional endpoints. Therefore, the available evidence supports an apoptosis-associated effect of 5-FU but does not establish a mitochondria-specific mechanism in this clinical setting.

In experimental GC models, hydrogen peroxide (H₂O₂) is commonly used as an experimental inducer of oxidative mitochondrial stress, causing ROS accumulation, mitochondrial dysfunction, and apoptosis 68. H₂O₂ should therefore be regarded as a mechanistic stressor rather than a therapeutic agent. Redox-modulating mitochondria-associated agents can also induce mitochondrial stress responses. For example, the nitric oxide-releasing prodrug NG increases intracellular ROS levels and induces mitochondrial dysfunction and apoptosis in MGC-803 cells, supporting its classification as a mitochondria-associated rather than mitochondria-targeted agent 69.

Several nonclassical anticancer agents also converge on mitochondrial pathways. Indomethacin induces mitochondrial apoptosis in GC cells by activating the PKCζ-p38-DRP1 signaling axis, thereby promoting mitochondrial fission and apoptotic signaling 70. Mito-FF, a mitochondria-targeting self-assembling peptide, disrupts mitochondrial structural integrity and induces profound mitochondrial collapse and apoptosis in GC models 71. In addition, the gold-based compound auranofin triggers GC cell apoptosis through ROS-mediated mitochondrial dysfunction and redox imbalance, further emphasizing the role of oxidative stress in mitochondria-centered therapeutic strategies 72, 73.

Collectively, despite differences in pharmacological class, these agents converge on mitochondrial dysfunction characterized by ROS dysregulation, mitochondrial membrane depolarization (ΔΨm loss), and activation of intrinsic apoptotic pathways. These convergent mitochondrial responses highlight mitochondria as a shared therapeutic vulnerability and a candidate target for further development of mitochondria-centered anticancer strategies in GC (Table 3). Notably, the majority of currently available evidence for these agents in GC remains limited to *in vitro* experimental models.

Because many mitochondria-associated therapeutic effects have been reported mainly in cell-based GC models, the interpretation of these findings requires standardized functional validation. Commonly used assays include mitochondrial membrane potential staining, mitochondrial ROS detection, oxygen consumption rate measurement, ATP quantification, mitochondrial morphology analysis, cytochrome c release, caspase activation, BCL-2 family protein assessment, cytosolic mtDNA detection, and apoptosis assays. However, these assays should be interpreted as mechanistic or preclinical evidence rather than direct proof of clinical efficacy. Differences in drug exposure, bioavailability, pharmacokinetics, tumor heterogeneity, tissue distribution, and systemic toxicity may limit the direct translation of *in vitro* findings into clinical benefit 74-78 (Table 2).

## Part III. Chinese Herbal and Natural Medicines Modulating Mitochondrial Dysfunction and Apoptosis in GC

Many natural compounds have been reported to suppress GC cell proliferation and survival through one or more mitochondria-associated mechanisms. Depending on the compound and study, reported effects include reactive oxygen species (ROS) accumulation, loss of mitochondrial membrane potential (ΔΨm), mitochondrial outer membrane permeabilization (MOMP), or downstream caspase activation. These findings suggest that mitochondrial dysfunction represents a potential therapeutic vulnerability that may be modulated by natural compounds (Table 4).

### 3.1. Flavonoids and Polyphenols as Mitochondrial Stress-Inducing Compounds in GC

Flavonoids and polyphenolic compounds constitute the most extensively investigated class of herbal-derived compounds associated with mitochondrial stress in GC, including baicalein, wogonin, apigenin, luteolin, curcumin, and resveratrol. In SGC-7901 cells, baicalein induced mitochondrial membrane depolarization and intrinsic apoptosis and also inhibited tumor growth in an SGC-7901 xenograft model 79. A separate study showed that baicalein enhanced cisplatin sensitivity in MGC-803, HGC-27, SGC-7901, and SGC-7901/DDP cells through apoptosis- and autophagy-related signaling 80. Luteolin impaired mitochondrial integrity, ETC activity, and ATP production in HGC-27, MFC, and MKN-45 cells, leading to intrinsic apoptosis 81; additional evidence implicates miR-34a/BCL-2 signaling 82.

Wogonin has been reported to suppress GC cell proliferation, invasion, and migration while inducing apoptosis. These effects have been associated with ROS accumulation and inhibition of Wnt/β-catenin and JAK-STAT3 signaling. However, direct mitochondrial functional endpoints, including mitochondrial membrane depolarization and MOMP, were not specifically evaluated in these studies 83, 84.

Apigenin has been associated with apoptosis and autophagic cell death in GC models. Available evidence implicates Akt/BCL-2-family signaling and, under hypoxic conditions, HIF-1α/EZH2-related endoplasmic reticulum stress and autophagy. However, direct mitochondrial functional endpoints such as ΔΨm and mitochondrial ROS were not established in the cited experimental study 85, 86.

In addition to flavonoids, polyphenolic compounds such as curcumin and resveratrol have been reported to suppress GC cell survival. Curcumin induces apoptosis and protective autophagy through PI3K/AKT/mTOR- and p53-associated signaling in SGC-7901, BGC-823, and MKN-28 cells; however, the cited studies did not directly measure mitochondrial ROS or ΔΨm 87, 88. Resveratrol promotes apoptosis through PI3K/AKT/p53- and NF-κB-related signaling in AGS, HGC-27, and SGC-7901 cells and has also shown activity in a GC xenograft model, but direct mitochondrial functional endpoints remain insufficiently characterized 89, 90. Collectively, these findings support apoptosis-modulating effects while indicating that the degree of direct mitochondrial involvement varies among compounds.

Recent evidence further extends the role of mitochondrial dysfunction beyond intrinsic apoptosis to immune modulation. Mitochondrial DNA released from damaged mitochondria can function as a danger-associated molecular pattern and activate innate immune signaling pathways, particularly the cGAS-STING axis. In GC models, isobavachalcone, a prenylated chalcone, has been reported to promote mitochondrial damage and mtDNA release through DHODH-related mitochondrial membrane remodeling, thereby activating STING-associated immune signaling and contributing to anti-GC effects. These findings suggest that mitochondria-associated natural compounds may influence not only cancer cell apoptosis but also tumor immune signaling. Nevertheless, because mtDNA-cGAS-STING signaling may exert context-dependent effects, including both immune activation and immune evasion, its clinical relevance in GC remains to be further validated 78, 91, 92.

### 3.2. Terpenoids Inducing Mitochondrial Oxidative Stress and Apoptosis

Terpenoid-derived natural compounds represent another important class of mitochondria-associated compounds in GC. Among these, triptolide and celastrol have been investigated for their ROS-associated and apoptosis-inducing effects.

Triptolide induces ROS accumulation in GC cells through direct inhibition of peroxiredoxin 2 (PRDX2), leading to endoplasmic reticulum stress, cytoprotective autophagy, and apoptosis 93. Additional evidence implicates MDM2/p53-related apoptotic regulation 94. Because direct mitochondrial functional endpoints were not consistently measured, triptolide should be regarded as a mitochondria-associated ROS-inducing compound rather than a directly validated mitochondria-targeted agent.

Similarly, celastrol has been reported to induce mitochondrial stress and cytotoxicity in preclinical GC models by directly inhibiting PRDX2, disrupting redox homeostasis, and promoting ROS-mediated mitochondrial dysfunction and apoptosis. These effects are accompanied by ΔΨm loss and have been observed in BGC-823 and SGC-7901 cells and an SGC-7901 xenograft model 95. Celastrol also suppresses prosurvival PI3K/AKT/NF-κB signaling 96. These findings support a mitochondria-associated, ROS-dependent mechanism, although translational development remains constrained by toxicity and pharmacokinetic concerns.

### 3.3. Quinone Derivatives as Potent Mitochondrial Stress Amplifiers

Quinone-derived natural compounds also exhibit strong mitochondria-modulating properties in GC. Shikonin, a naphthoquinone compound isolated from Lithospermum erythrorhizon, demonstrates potent prooxidant activity in GC cells. Experimental studies show that shikonin rapidly induces ROS accumulation, mitochondrial membrane depolarization, mitochondrial outer membrane permeabilization, and caspase-dependent apoptosis in human GC cells 97, 98. These mitochondrial alterations highlight the sensitivity of GC mitochondria to oxidative stress and identify shikonin as an experimental inducer of mitochondrial apoptosis.

### 3.4. Alkaloids Targeting Mitochondria-Dependent Apoptosis

Alkaloid-derived natural compounds also contribute to apoptosis-related anticancer mechanisms in GC. Matrine, a quinolizidine alkaloid isolated from Sophora flavescens, induces BCL-2-family- and caspase-dependent apoptosis in MKN-45 cells and inhibits BGC-823 xenograft growth. The cited studies support apoptosis-related activity but do not directly establish ROS accumulation or ΔΨm loss 99, 100.

Despite encouraging experimental findings, many natural compounds such as curcumin, baicalein, and resveratrol exhibit limited bioavailability, complex pharmacokinetic behavior, and uncertain clinically achievable concentrations *in vivo*. Curcumin, for example, is characterized by poor aqueous solubility, limited intestinal absorption, rapid metabolism, and rapid systemic clearance, which may restrict its effective plasma and tissue concentrations after conventional administration 101. Similarly, baicalein and related flavonoids may show limited oral bioavailability because of poor solubility, extensive metabolism, and absorption-related barriers 102. Therefore, mitochondrial effects observed *in vitro* may not be directly achievable *in vivo* at comparable concentrations. Potential systemic toxicity also requires dedicated pharmacological evaluation. Without optimized delivery systems, such as nanoformulations, liposomal carriers, phospholipid complexes, or other tumor-targeted delivery strategies, the translational potential of these natural compounds as primary mitochondria-centered therapies for GC remains preliminary and speculative. Further pharmacokinetic optimization, standardized formulations, *in vivo* validation, and well-designed clinical studies are required before these compounds can be considered clinically applicable mitochondria-centered therapeutic strategies for GC.

Taken together, diverse classes of herbal-derived compounds—including flavonoids, polyphenols, terpenoids, quinone derivatives, and alkaloids—can converge on apoptosis-related and, in selected studies, directly measured mitochondrial pathways. However, most available evidence remains preclinical, particularly *in vitro*. Depending on the compound and experimental endpoints, reported effects include ROS accumulation, mitochondrial membrane depolarization, and activation of intrinsic apoptotic cascades. These findings identify mitochondrial dysfunction as a candidate vulnerability warranting further validation in GC (Table 4).

Although many natural compounds have shown encouraging mitochondria-associated anticancer effects in GC models, their clinical translation remains limited by several important factors. Many phytochemicals exhibit poor aqueous solubility, low oral bioavailability, rapid metabolism, and uncertain pharmacokinetic profiles, which may restrict their effective concentrations in tumor tissues 103, 104. In addition, the mitochondrial effects observed *in vitro* are often dose-dependent and may not be clinically achievable *in vivo*. Potential safety concerns, including off-target toxicity, herb-drug interactions, and tissue-specific mitochondrial stress, also require careful evaluation. Therefore, further studies using standardized formulations, pharmacokinetic assessment, animal models, and well-designed clinical trials are needed before these compounds can be considered for clinical application in GC, particularly in the context of precision medicine-based mitochondrial targeting strategies.

Overall, while natural compounds provide valuable mechanistic insights into mitochondrial vulnerability in GC, their clinical applicability remains to be rigorously validated.

### Part IV. Future Challenges and Perspectives of Mitochondria-Targeted Therapy in GC

Although extensive *in vitro* studies suggest that mitochondrial dysfunction represents an important therapeutic vulnerability in GC, robust translational validation in *in vivo* models and clinical settings remains limited. Importantly, to date, there is no definitive prospective clinical evidence demonstrating that direct targeting of mitochondrial dysfunction alone improves survival outcomes in patients with GC. Therefore, mitochondrial dysfunction should be regarded as a biologically plausible and experimentally supported candidate vulnerability rather than an established clinically validated therapeutic target. Most mitochondria-targeted agents investigated in GC have been evaluated primarily in cell-based experimental systems, whereas comparatively few studies have incorporated direct mitochondrial functional assessments—such as mitochondrial membrane potential (ΔΨm), mitochondrial dynamics, or mitophagy flux—in animal models.

Among currently available examples, HSP90 inhibitors such as 17-DMAG have demonstrated antitumor activity in GC xenograft models. These effects are accompanied by increased expression of intrinsic apoptotic markers, including altered BAX/BCL-2 ratios, cytochrome c release, and activation of caspase-9 and caspase-3 signaling pathways. However, most *in vivo* investigations primarily focus on apoptosis-related surrogate endpoints rather than direct measurements of mitochondrial functional alterations. This discrepancy highlights a critical gap between mechanistic insights derived from *in vitro* studies and their translational validation in more physiologically relevant *in vivo* models and clinical contexts 62.

In parallel, mitochondria-targeted delivery strategies have begun to demonstrate encouraging preclinical activity. For example, the self-assembling peptide Mito-FF has been reported to exhibit enhanced antitumor activity in GC xenograft models through preferential mitochondrial accumulation and amplification of mitochondrial oxidative stress and apoptosis-associated signaling pathways. These findings support further investigation of mitochondria-directed therapeutic strategies *in vivo* and underscore the importance of integrating mitochondrial biology into translational study design 71.

Looking forward, future research should prioritize the following directions:

(i) Incorporation of GC-relevant *in vivo* models with direct mitochondrial functional measurements;

(ii) Identification of mitochondria-based pharmacodynamic biomarkers that can be applied to clinical specimens;

(iii) Development of rational combination strategies integrating mitochondrial targeting with conventional chemotherapy or molecular-targeted therapies.

### Rational combination strategies

Based on the mitochondrial targets and compound classes summarized in this review, several rational combination strategies may be considered. First, conventional chemotherapeutic agents may be combined with mitochondria-associated apoptosis sensitizers to lower the apoptotic threshold of GC cells, particularly in tumors with dysregulated BCL-2 family proteins or impaired mitochondria-dependent apoptosis 35, 36, 62, 67, 71-73. Second, ROS-inducing natural compounds may be combined with agents that impair antioxidant defense or redox homeostasis, although systemic toxicity and tissue-specific mitochondrial injury must be carefully evaluated 79-104. Third, metabolic modulators targeting glycolysis-OXPHOS plasticity, such as the HK2-VDAC axis or PDK-PDH metabolic gate, may be explored in combination with chemotherapy to overcome metabolic adaptation and therapeutic resistance 31, 34, 42, 43, 45-47. Fourth, mitochondrial stress-inducing agents that promote mtDNA release or cGAS-STING activation may provide a rationale for combination with immune-modulating strategies; however, this approach requires careful evaluation because mtDNA-cGAS-STING signaling can exert context-dependent effects, including both immune activation and immune evasion 78, 91, 92. Finally, biomarker-guided stratification using mitochondrial metabolic markers, BCL-2 family proteins, HK2, VDAC1, SIRT3, or mtDNA-related immune markers may help identify patient subgroups more likely to benefit from mitochondria-centered combinations 31-36, 42, 43, 78, 91, 92. These strategies remain hypothesis-generating and should be validated in organoids, xenograft models, patient-derived xenografts, pharmacokinetic studies, safety assessments, and prospective clinical trials before clinical recommendation 23, 24, 105.

Addressing these challenges will be essential for translating mitochondria-centered concepts from experimental systems into clinically relevant therapeutic strategies for GC 23, 24, 105.

Although numerous mitochondrial biomarkers and mitochondria-modulating compounds have shown encouraging preclinical results, most remain at an early stage of development and have not yet undergone adequate clinical validation. Future studies integrating mitochondrial biomarkers with clinical stratification strategies and well-designed clinical trials will be essential to support future clinical translation of mitochondria-centered therapeutic strategies.

## Conclusions

Mitochondrial dysfunction has emerged as a central hallmark of GC, mechanistically linking metabolic reprogramming, oxidative stress imbalance, dysregulated mitochondrial dynamics, and evasion of apoptosis to tumor initiation, progression, and therapeutic resistance. Accumulating evidence indicates that alterations in glycolysis-mitochondrial coupling, remodeling of the electron transport chain (ETC)/oxidative phosphorylation (OXPHOS) system, redox homeostasis, mitochondrial quality control, and mitochondrial genome stability collectively contribute to the metabolic plasticity and aggressive clinical behavior of GC. Importantly, many of these mitochondrial alterations have been associated with poor clinical outcomes and chemotherapeutic resistance, highlighting their potential value as both prognostic biomarkers and mechanistically informed therapeutic targets.

Beyond their role in tumorigenesis, mitochondrial vulnerabilities may represent candidate therapeutic opportunities in anti-GC therapy, as mitochondrial metabolism has emerged as an attractive therapeutic target in multiple cancer types 24. Both conventional chemotherapeutic agents and emerging mitochondria-targeted and mitochondria-modulating strategies—including small molecules and herbal-derived natural compounds—appear to converge on several common mitochondrial outcomes, such as reactive oxygen species (ROS) dysregulation, mitochondrial membrane depolarization, mitochondrial outer membrane permeabilization (MOMP), and activation of intrinsic apoptotic signaling pathways. Although translational evidence remains relatively limited, particularly regarding direct mitochondrial functional measurements in *in vivo* models, current data support the further exploration of mitochondrial dysfunction as a candidate therapeutic vulnerability rather than an established clinical therapeutic target.

Collectively, these findings suggest that mitochondria function not merely as passive bystanders of metabolic reprogramming but as active regulators of tumor behavior and therapeutic response in GC. Future investigations integrating GC-relevant *in vivo* models, refined mitochondrial biomarkers, and rational combination strategies will be essential for advancing mitochondria-centered precision therapeutic approaches in GC. As a mechanistically oriented narrative review, this work provides an integrated framework for understanding how mitochondrial dysfunction can be further explored for biomarker development and therapeutic intervention in GC.

## Figures and Tables

**Table 1:** Mitochondrial biomarkers associated with metabolic reprogramming, oxidative stress regulation, mitochondrial dynamics, and mitochondria-dependent apoptosis in GC.

Category	Biomarker	Canonical Function	Role in GC	Expression/Localization	Sample	Detection	Clinical Significance	Evidence Level	Ref.
Reprogrammed glycolysis (Warburg effect)	HK2	Catalyzes glucose → glucose-6-phosphate	Promotes aerobic glycolysis; binds VDAC to inhibit mitochondrial apoptosis	High (OMM, cytosol, MAMs)	Tissue	IHC, WB	Poor prognosis; reduced OS	CL	31, 34
	VDAC1	OMM metabolite channel	HK2-VDAC interaction suppresses apoptosis	Expressed (OMM)	Tissue	IHC	Mechanistically linked to apoptosis resistance; independent prognostic value not established	IVT, CL	34, 42, 43
Altered mitochondrial metabolic enzymes	PDK1	PDH kinase; inhibits pyruvate oxidation	Blocks pyruvate entry into TCA cycle and promotes glycolytic shift	High (mitochondrial matrix)	Tissue	IHC, qPCR	Poor prognosis	IVT, CL	45
	PDK4	PDH kinase; inhibits pyruvate oxidation	Blocks pyruvate entry into TCA cycle and promotes glycolytic shift	Overall downregulated/dysregulated in GC datasets	TCGA-STAD and GSE54129 datasets	RNA-seq and bioinformatics analysis	Context-dependent associations with tumor stage and survival; further validation required	CL	47
	PDH	Links glycolysis to TCA cycle	Reduced acetyl-CoA production and OXPHOS activity	Low (mitochondrial matrix)	Tissue	IHC	Poor prognosis	CL	46
ETC/TCA axis	NDUFA4	Mitochondrial complex IV-associated protein	Promotes glycolytic and oxidative metabolism and cell proliferation	High (IMM)	Tissue	IHC	Poor prognosis	IVT, IVV, CL	48, 49
	FH (tissue)	TCA cycle enzyme (fumarate → malate)	FH suppression alters fumarate metabolism and increases cisplatin sensitivity	FH expression/activity (matrix/cytosol)	GC tissue/cell models	IHC, WB, FH activity assay	Treatment-response relevance; prognostic value not established	IVT	50
	FH-Ab (serum)	Circulating autoantibody against FH	Stage-specific serum immune signature	Reduced serum FH-Ab levels in advanced disease	Serum	ELISA	Lower levels associated with poorer outcomes and shorter survival	CL	**38**
	PGC-1α	Regulator of mitochondrial biogenesis	Controls mitochondrial transcription and oxidative metabolism	Context-dependent (nucleus)	Literature-derived evidence	Not standardized	GC-specific prognostic value not established	Review	51
Altered oxidative stress regulation	SIRT3	Mitochondrial deacetylase	Maintains redox homeostasis; loss increases mtROS	Low (mitochondrial matrix)	Tissue	IHC	Poor prognosis	CL	32, 33
	SOD2	Mitochondrial superoxide detoxification enzyme	Regulates mitochondrial oxidative stress	Altered (mitochondrial matrix)	Tissue/GC models	IHC, WB	Associated with invasion; prognostic value requires validation	IVT, CL	52, 53
	mtGSH	Mitochondrial antioxidant pool	Maintains mitochondrial redox balance	Mitochondrial matrix pool; GC-specific pattern not established	Experimental models	Biochemical assays	Mechanistic relevance; GC-specific clinical significance not established	Review	54
Altered mitochondrial dynamics/quality control	DRP1	Mitochondrial fission GTPase	Promotes mitochondrial fission and treatment resistance in GC cell models	Activated/recruited to OMM	GC cell models	WB, mitochondrial morphology assays	Associated with adriamycin resistance; clinical significance not established	IVT	55, 56
	PINK1/Parkin	Mitophagy-initiating kinase/E3 ubiquitin ligase	Context-dependent regulation of mitophagy, cisplatin resistance, and hypoxia-related aggressiveness	Context-dependent pathway activity (OMM/cytosol)	GC cell models	WB; mitophagy and mtROS assays	Clinical significance not established	IVT	57, 58
	TFAM	mtDNA transcription factor	TFAM knockdown reduces mtDNA copy number and respiration and promotes glycolytic reprogramming and cell migration	Experimentally reduced	AGS cells	qPCR, WB, mtDNA copy-number analysis, OCR	Clinical prognostic value of TFAM expression not established	IVT	59
Mitochondria-dependent apoptosis	BCL-2	Anti-apoptotic protein	Blocks cytochrome c release and contributes to altered chemosensitivity	Variable (OMM)	Tissue/GC models	IHC, WB	Chemosensitivity relevance; prognostic association inconsistent	IVT, CL	35-37

**Note:** This table summarizes representative mitochondrial-associated biomarkers involved in metabolic reprogramming, mitochondrial metabolism, oxidative stress regulation, mitochondrial dynamics, mitochondrial quality control, and mitochondria-dependent apoptosis in GC. Depending on the biomarker, evidence ranges from mechanistic preclinical studies to clinical association studies; not all entries have validated prognostic significance in GC. Evidence Level: IVT = *in vitro* study; IVV = *in vivo* animal model; CL = clinical evidence; Review = evidence derived primarily from review literature. **Abbreviations:** OMM: outer mitochondrial membrane; IMM: inner mitochondrial membrane; MAMs: mitochondria-associated membranes; TCA: tricarboxylic acid cycle; ETC: electron transport chain; OXPHOS: oxidative phosphorylation; mtROS: mitochondrial reactive oxygen species; mtGSH: mitochondrial glutathione; mtDNA: mitochondrial DNA; IHC: immunohistochemistry; WB: western blot; qPCR: quantitative polymerase chain reaction; ELISA: enzyme-linked immunosorbent assay; OCR: oxygen consumption rate; OS: overall survival; FH: fumarate hydratase; FH-Ab: fumarate hydratase autoantibody; TCGA-STAD: The Cancer Genome Atlas stomach adenocarcinoma cohort; GSE54129: Gene Expression Omnibus dataset GSE54129; RNA-seq: RNA sequencing.

**Table 2:** Experimental methods for evaluating mitochondria-related functional effects in GC models.

Mitochondrial effect	Common Detection Methods	Biological Interpretation	Ref.
Mitochondrial membrane potential loss	JC-1, TMRE, or TMRM staining	Indicates mitochondrial depolarization and early mitochondrial injury	74
ROS generation	MitoSOX staining for mitochondrial superoxide; DCFH-DA staining for general intracellular ROS	Assesses mitochondrial superoxide or general oxidative stress, depending on the probe	74
OXPHOS impairment	Seahorse OCR assay, ETC complex activity assay	Indicates reduced mitochondrial respiration	74, 75
ATP depletion	Luminescence-based ATP assay	Suggests impaired mitochondrial energy production	74
Mitochondrial morphology change	MitoTracker staining, TEM, or confocal microscopy	Reflects mitochondrial fission, fragmentation, or swelling	76
Cytochrome c release	WB or IF	Indicates mitochondrial outer membrane permeabilization	74
Caspase activation	Caspase-9/3 activity assay, cleaved caspase-3 WB	Confirms intrinsic apoptosis activation	74
BCL-2 family modulation	WB, qPCR, or IHC	Reflects altered apoptotic threshold	74
mtDNA release	Cytosolic mtDNA qPCR; cGAS-STING pathway markers	Indicates mitochondrial damage and potential immune activation	77, 78
Cell death/apoptosis	Annexin V/PI staining, TUNEL assay, or flow cytometry	Confirms downstream cell death outcome	74

**Abbreviations:** ATP: adenosine triphosphate; BCL-2: B-cell lymphoma 2; cGAS: cyclic GMP-AMP synthase; DCFH-DA: 2′,7′-dichlorodihydrofluorescein diacetate; ETC: electron transport chain; IF: immunofluorescence; IHC: immunohistochemistry; JC-1: 5,5′,6,6′-tetrachloro-1,1′,3,3′-tetraethylbenzimidazolylcarbocyanine iodide; mtDNA: mitochondrial DNA; OCR: oxygen consumption rate; OXPHOS: oxidative phosphorylation; PI: propidium iodide; qPCR: quantitative polymerase chain reaction; ROS: reactive oxygen species; STING: stimulator of interferon genes; TEM: transmission electron microscopy; TMRE: tetramethylrhodamine ethyl ester; TMRM: tetramethylrhodamine methyl ester; TUNEL: terminal deoxynucleotidyl transferase dUTP nick-end labeling; WB: western blot; ΔΨm: mitochondrial membrane potential. These assays are commonly used to evaluate mitochondrial function, mitochondrial stress, intrinsic apoptosis, and mtDNA-related innate immune activation in cellular or preclinical models. The methodological rationale was summarized based on established mitochondrial function assessment guidelines and mtDNA-cGAS-STING detection protocols 74-78.

**Table 3:** Representative mitochondria-associated agents, mitochondria-targeted systems, and experimental interventions affecting mitochondrial function and/or apoptosis in GC models.

Agent	Drug Class	Mitochondrial Mechanism	Key Mitochondrial Effects	Experimental Model	GC Cell Lines/ Model	Evidence Level	Ref.
17-DMAG	HSP90 inhibitor	Oxidant-antioxidant imbalance and apoptosis	ROS ↑, antioxidant defenses ↓, apoptosis	*In vitro* *In vivo*	AGS	IVT IVV	62
DOX + Parameritannin A-2	Topoisomerase II inhibitor	DOX-associated mitochondrial apoptosis	ROS ↑, MOMP, ΔΨm loss	*In vitro*	HGC-27	IVT	63
PDOX	Cathepsin B-cleavable DOX prodrug	Mitochondria-associated oxidative stress and apoptosis	ROS ↑, mitochondrial apoptosis	*In vitro*	MGC-803	IVT	64
Topotecan (TPT)	Topoisomerase I inhibitor	Mitochondrial apoptotic activation	ROS ↑, ΔΨm loss	*In vitro*	AGS BGC-823	IVT	65, 66
5-Fluorouracil (5-FU)	Pyrimidine analogue	Apoptosis-associated effect; mitochondria-specific mechanism not directly evaluated	Increased apoptotic fraction	*Clinical tissue study*	Patients with advanced GC	CL	67
H₂O₂	ROS inducer	Oxidative mitochondrial damage	ROS ↑, ΔΨm loss	*In vitro*	MGC-803	IVT	68
NG	Nitric oxide-releasing prodrug	Mitochondrial oxidative stress	ROS ↑, mitochondrial apoptosis	*In vitro*	MGC-803	IVT	69
Indomethacin	Cyclooxygenase inhibitor	Mitochondrial apoptosis activation	ΔΨm loss, apoptosis	*In vitro*	AGS KATO III	IVT	70
Mito-FF	Mitochondria-targeting peptide	Direct mitochondrial disruption	ROS ↑, mitochondrial apoptosis	*In vitro* *In vivo*	AGS cells and AGS xenografts in nude mice	IVT IVV	71
Auranofin (AF)	Thioredoxin reductase inhibitor	Redox disruption and mitochondrial stress	ROS ↑, ΔΨm loss	*In vitro*	BGC-823 SGC-7901	IVT	72, 73

**Note:** This table summarizes representative pharmacological agents, mitochondria-directed systems, and experimental interventions reported to induce mitochondrial dysfunction and mitochondria-dependent apoptosis in GC experimental models. The observed mitochondrial effects include oxidative stress, loss of mitochondrial membrane potential (ΔΨm), mitochondrial outer membrane permeabilization (MOMP), cytochrome c release, and activation of intrinsic apoptosis. Most available evidence remains limited to *in vitro* studies and should therefore be interpreted as preclinical mechanistic evidence rather than proof of clinical efficacy. Only compounds or delivery systems specifically designed to accumulate within mitochondria or directly disrupt mitochondrial structures or functions are classified as mitochondria-targeted agents; Mito-FF is a representative example. Most conventional pharmacological agents listed in this table are classified as mitochondria-associated agents because their primary targets are not mitochondrial, although they secondarily induce mitochondrial stress. H₂O₂ is included solely as an experimental inducer of oxidative mitochondrial stress and should not be interpreted as a therapeutic agent. **Evidence Level: IVT, *in vitro* study; IVV, *in vivo* animal model; CL, clinical evidence.** **Abbreviations:** ROS: reactive oxygen species; ΔΨm: mitochondrial membrane potential; MOMP: mitochondrial outer membrane permeabilization; DOX: doxorubicin; PDOX: Ac-Phe-Lys-PABC-doxorubicin prodrug; TPT: topotecan; 5-FU: 5-fluorouracil; NG: nitric oxide-releasing prodrug; AF: auranofin; GC: gastric cancer.

**Table 4:** Chinese herbal and natural compounds modulating mitochondrial pathways in GC experimental models.

Natural compound (representative botanical source)	Phytochemical Class	Mitochondrial Mechanism	Key Mitochondrial Effects	Experimental Model	GC cell lines and animal models	Evidence Level	Ref.
Baicalein (*Scutellaria baicalensis*)	Flavonoid	Induces mitochondrial apoptosis and enhances cisplatin sensitivity through apoptosis/autophagy-related signaling	ΔΨm loss, BAX/BCL-2 modulation, apoptosis, autophagy	*In vitroIn vivo*	SGC-7901, MGC-803, HGC-27, SGC-7901/DDP; SGC-7901 xenograft	IVT,IVV	79, 80
Luteolin (*Lonicera japonica*)	Flavonoid	Impairs mitochondrial integrity and ETC activity, activating intrinsic apoptosis	ΔΨm loss, ETC complex I/III/V activity ↓, ATP depletion, apoptosis	*In vitro*	HGC-27, MFC, MKN-45	IVT	81, 82
Isobavachalcone* (Psoralea corylifolia)*	Prenylated chalcone/flavonoid-related compound	Promotes mitochondrial damage and mtDNA release through DHODH-related mitochondrial membrane remodeling; activates STING-related immune signaling	ROS ↑, mtDNA release, STING activation, immune modulation	*In vitro* *In vivo*	HGC-27 and SNU-719 cells; BALB/c mouse model	IVT, IVV	78, 91, 92
Wogonin (*Scutellaria baicalensis*)	Flavonoid	ROS-associated apoptosis with inhibition of Wnt/β-catenin and JAK-STAT3 signaling	ROS ↑, apoptosis; mitochondrial functional endpoints not directly evaluated	*In vitro* *In vivo*	SGC-7901 and BGC-823 cells; SGC-7901 xenograft	IVT, IVV	83, 84
Apigenin (*Apium graveole*ns)	Flavonoid	Promotes apoptosis and autophagic cell death through Akt/BCL-2-family and HIF-1α/EZH2/ER-stress pathways; direct mitochondrial functional endpoints incompletely characterized	Apoptosis, autophagy; direct ΔΨm and mitochondrial ROS endpoints not established in the cited experimental study	*Review* *In vitro*	AGS, SNU-638	Review, IVT	85, 86
Curcumin (*Curcuma longa*)	Polyphenol	Induces apoptosis and protective autophagy through PI3K/AKT/mTOR and p53 signaling; direct mitochondrial functional endpoints were not evaluated	Apoptosis, autophagy; direct ΔΨm and mitochondrial ROS endpoints not reported	*In vitro*	SGC-7901, BGC-823, MKN-28	IVT	87, 88
Resveratrol (*Polygonum cuspidatum*)	Polyphenol	Induces apoptosis through PI3K/AKT/p53- and NF-κB-related signaling; direct mitochondrial functional endpoints were not evaluated	Apoptosis and cell-cycle arrest; direct ΔΨm and mitochondrial ROS endpoints not reported	*In vitroIn vivo*	AGS, HGC-27, SGC-7901; GC xenograft model	IVT,IVV	89, 90
Triptolide (*Tripterygium wilfordii*)	Terpenoid	Induces PRDX2-dependent ROS accumulation, ER stress, cytoprotective autophagy, and apoptosis	ROS ↑, ER stress, apoptosis, autophagy; direct ΔΨm endpoint not established	*In vitro*	AGS, SGC-7901	IVT	93, 94
Celastrol (*Tripterygium wilfordii*)	Terpenoid	Induces PRDX2-dependent ROS accumulation, mitochondrial dysfunction, and apoptosis; also suppresses PI3K/AKT/NF-κB signaling	ROS ↑, ΔΨm loss, mitochondrial dysfunction, apoptosis	*In vitroIn vivo*	BGC-823, SGC-7901; SGC-7901 xenograft	IVT,IVV	95, 96
Matrine (*Sophora flavescens*)	Alkaloid	Induces BCL-2-family- and caspase-dependent apoptosis	BAX/BCL-2 modulation, caspase activation, apoptosis; direct ROS and ΔΨm endpoints not established	*In vitroIn vivo*	MKN-45; BGC-823 xenograft in nude mice	IVT,IVV	99, 100
Shikonin (*Lithospermum erythrorhizon*)	Naphthoquinone	Induces mitochondrial oxidative stress and apoptosis	ROS ↑, ΔΨm loss, mitochondrial apoptosis	*In vitro*	HGC-27, AGS, MKN-45	IVT	97, 98

**Note:** This table summarizes representative natural compounds reported to affect apoptosis-related or mitochondrial pathways in GC experimental models. The degree of direct mitochondrial validation varies substantially among compounds: some studies measured mitochondrial functional endpoints, whereas others evaluated ROS, apoptosis, autophagy, or signaling pathways without directly assessing mitochondrial ROS, ΔΨm, MOMP, or respiration. Most evidence remains preclinical, and only a minority of compounds have *in vivo* validation. Bioavailability, pharmacokinetics, formulation variability, and systemic toxicity should therefore be considered when interpreting translational potential. These compounds should be regarded as mitochondria-associated or apoptosis-modulating agents with preliminary evidence rather than clinically validated mitochondria-targeted therapies. Evidence Level: IVT: *in vitro* study; IVV: *in vivo* animal model; CL: clinical evidence; Review: evidence derived primarily from review literature. **Abbreviations:** ROS: reactive oxygen species; ΔΨm: mitochondrial membrane potential; MOMP: mitochondrial outer membrane permeabilization; ETC: electron transport chain; ATP: adenosine triphosphate; BAX: BCL-2-associated X protein; BCL-2: B-cell lymphoma 2; PRDX2: peroxiredoxin 2; ER: endoplasmic reticulum; GC: gastric cancer.

## Abbreviations

17-DMAG: 17-dimethylaminoethylamino-17-demethoxygeldanamycin
5-FU: 5-fluorouracil
AF: auranofin
AGS: human gastric adenocarcinoma cell line
AKT: protein kinase B
AMP: adenosine monophosphate
AMPK: AMP-activated protein kinase
ATP: adenosine triphosphate
BATF2: basic leucine zipper ATF-like transcription factor 2
BAX: BCL-2-associated X protein
BCL-2: B-cell lymphoma 2
BRCA1/2: breast cancer susceptibility genes 1 and 2
cGAS: cyclic GMP-AMP synthase
CL: clinical evidence
CLDN18.2: claudin 18 isoform 2
DCFH-DA: 2',7'-dichlorodihydrofluorescein diacetate
DHODH: dihydroorotate dehydrogenase
DNM1L: dynamin 1-like protein
DOX: doxorubicin
DRP1: dynamin-related protein 1
EGFR: epidermal growth factor receptor
ELISA: enzyme-linked immunosorbent assay
ER: endoplasmic reticulum
ERK1/2: extracellular signal-regulated kinases 1 and 2
ETC: electron transport chain
FH: fumarate hydratase
FH-Ab: fumarate hydratase autoantibody
FIS1: mitochondrial fission 1 protein
GC: gastric cancer
HER2: human epidermal growth factor receptor 2
HIF-1α: hypoxia-inducible factor 1 alpha
HK2: hexokinase 2
HSP90: heat shock protein 90
IF: immunofluorescence
IHC: immunohistochemistry
IMM: inner mitochondrial membrane
IVT: *in vitro* study
IVV: *in vivo* animal model
JC-1: 5,5',6,6'-tetrachloro-1,1',3,3'-tetraethylbenzimidazolylcarbocyanine iodide
KRAS: KRAS proto-oncogene GTPase
LKB1: liver kinase B1
MAMs: mitochondria-associated membranes
MFC: murine forestomach carcinoma cell line
MFF: mitochondrial fission factor
MID49: mitochondrial dynamics protein of 49 kDa
MID51: mitochondrial dynamics protein of 51 kDa
MMR: mismatch repair
MOMP: mitochondrial outer membrane permeabilization
MSI: microsatellite instability
mtDNA: mitochondrial DNA
mtGSH: mitochondrial glutathione
mtROS: mitochondrial reactive oxygen species
MYC: MYC proto-oncogene
NAD+: oxidized nicotinamide adenine dinucleotide
NADH: reduced nicotinamide adenine dinucleotide
NDUFA4: NDUFA4 mitochondrial complex-associated protein
NF-κB: nuclear factor kappa B
NG: nitric oxide-releasing prodrug
NRF1/2: nuclear respiratory factors 1 and 2
OCR: oxygen consumption rate
OMM: outer mitochondrial membrane
OS: overall survival
OXPHOS: oxidative phosphorylation
PARK2: parkin RBR E3 ubiquitin protein ligase
PD-L1: programmed death-ligand 1
PDOX: Ac-Phe-Lys-PABC-doxorubicin prodrug
PDH: pyruvate dehydrogenase
PDHA1: pyruvate dehydrogenase E1 subunit alpha 1
PDK: pyruvate dehydrogenase kinase
PDK1: pyruvate dehydrogenase kinase 1
PDK4: pyruvate dehydrogenase kinase 4
PGC-1α: peroxisome proliferator-activated receptor gamma coactivator 1 alpha
PI: propidium iodide
PI3K: phosphoinositide 3-kinase
PINK1: PTEN-induced kinase 1
PKCζ: protein kinase C zeta
PRDX2: peroxiredoxin 2
PTEN: phosphatase and tensin homolog
qPCR: quantitative polymerase chain reaction
RB1: retinoblastoma 1
ROS: reactive oxygen species
SIRT3: sirtuin 3
SOD2: superoxide dismutase 2
STAT3: signal transducer and activator of transcription 3
STING: stimulator of interferon genes
TCA: tricarboxylic acid cycle
TEM: transmission electron microscopy
TFAM: mitochondrial transcription factor A
TMRE: tetramethylrhodamine ethyl ester
TMRM: tetramethylrhodamine methyl ester
TPT: topotecan
TUNEL: terminal deoxynucleotidyl transferase dUTP nick-end labeling
VDAC: voltage-dependent anion channel
VDAC1: voltage-dependent anion channel 1
VEGF: vascular endothelial growth factor
WB: western blot
ΔΨm: mitochondrial membrane potential
